# Retraction notice for: “lncRNA CCAT1 promotes cell proliferation, migration, and invasion by down-regulation of miR-143 in FTC-133 thyroid carcinoma cell line” [Braz J Med Biol Res (2018) 51(6): e7046]

**DOI:** 10.1590/1414-431X2020e7046retraction

**Published:** 2021-06-14

Tianzheng Yang^1^, Hongyan Zhai^1^, Ruihong Yan^1^, Zhenhu Zhou^1^, Lei Gao^1^, Luqing Wang^2^

^1^Department of Nuclear Medicine, Liaocheng People’s Hospital, Liaocheng, Shandong, China

^2^Department of Radioimmunoassay, Liaocheng People’s Hospital, Liaocheng, Shandong, China

**Retraction for:** Braz J Med Biol Res | doi: http://10.1590/1414-431x20187046 | PMID: 29791590 | PMCID: PMC6002139

The authors would like to retract the article “lncRNA CCAT1 promotes cell proliferation, migration, and invasion by down-regulation of miR-143 in FTC-133 thyroid carcinoma cell line” that was published in volume 51 no. 6 (2018) (Epub May 21, 2018) in the Brazilian Journal of Medical and Biological Research.

After the publication of this study, the corresponding author requested its retraction due to “the identification of data fabrication.” The Editors decided to immediately retract this paper to avoid further damage to the scientific community.

The Brazilian Journal of Medical and Biological Research remains vigilant to prevent misconduct and reinforces the Journal’s commitment to good scientific practices. We regret the unprofessional behavior of the authors involved.

Correspondence: Ruihong Yan: <hongruiyan001@163.com> (new email)

